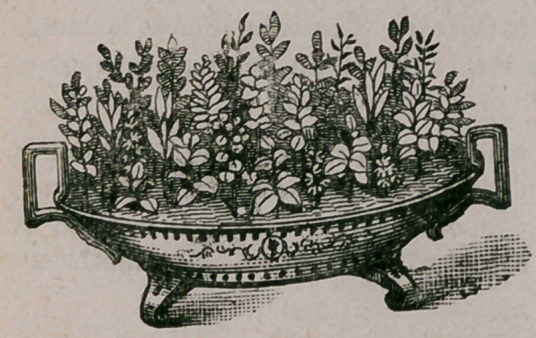# Household

**Published:** 1888-07

**Authors:** 


### HOUSEHOLD.

### THE MUD SYSTEM OF SLIPPING PLANTS.

A child of five years can cut off a slip from a geranium, verbena, heliotrope, carnation, fuchsia, or even a rosebush, taking care that the slip is made from the young or green shoot ; and in a plate or saucer filled with wet sand it will root just as quickly and as well as if put in by the hands of a gardener—provided care is taken that the sand in the saucer is kept wet by adding a little water to it each day until the slips show the small roots. The slip should be cut in the way shown in the drawing, taking it off either between or below the joints. The saucer holding the slips should be placed in some sunny window where it is warm. Nearly all kinds of slips can be rooted at any time of the year ; but some, such as the coleus, salvias, and various plants called "warm-blooded," had better not be slipped until the warm weather comes in May.

The slips will begin to show little roots in from two to three weeks after being put in the saucers. They should then be potted in little pots about two inches deep, which the gardeners call thumb-pots, in rich soft mold, which can be procured from any florist. Good garden earth will also do, only it must not be wet and sticky. If it can only be got in a very wet condition, dry stove-ashes may be mixed with it.

When the slips are to be potted, first fill the little flower-pot full of earth, then with the fore-finger make a hole in the center big enough to put the roots in. Gently press the earth all around the roots, making it level and smooth on the top ; then with a watering-pot sprinkle slightly the slips, now plants. Every other day they will require watering until they begin to put little white roots to the edge of the pot, which can be seen by giving the pot a tap on the table, and turning the contents out just like jelly from a glass. After the soil in the little pots gets filled with roots, which will be in four or five weeks from the time the slips were placed in them, it will be well to transplant into pots three or four inches deep. In a few weeks the slips will have made plants large enough to set out in the open garden, and by midsummer will be fine bushes covered with blossoms,

### HINTS FOR WINTER GARDENS.

CINERARIAS.—Few pot-plants present a more attractive appearance in late winter and early spring than do the cineraries when at the height of their beauty, bearing, as they do, immense clusters of bright, handsome flowers boldly above the bright leaves. They are capital plants for a cool greenhouse, or, with care, in a window, and are raised from seeds sown annually at any time from July to September. The seeds are fine, and require careful treatment in sowing, to begin with. As soon as the young seedings can be handled, they should be potted into small pots, and given a place near the glass. A low sash frame that is covered with shaded glass in a good place for them until Oct. 1. Always shift the plants into larger pots before the roots mat around the ball of earth, as their growth is liable to be cheeked most unfavorably if they become pot-bound. The plants like a rich soil abounding in sand and vegetable fibre.

CASEMENT WINDOW CURTAINS.—The difficulty of curtaining casement windows which open inward is well-known. This difficulty is obviated by a novel and very decorative treatment. A canopy frame is made, consisting of a semicircular rod, or bar of wood or metal, flatly covered over the top, and jutting out from the top of the window into the room. A valance or fringe encircles this bar, of material to match the curtains, which are hung underneath by brass rings—so that they may be drawn back at will. Windows treated in this way have a charming effect.

BRIGHT SILVER.—In a pantry where the mistress presides, a very simple method of cleaning silver may be adopted. After the breakfast dishes have been washed and put away, gather together all the silver, and put it to soak in a dish-pan of hot water, in which has been dissolved a handful of borax and a small bit of soup. During all the morning while about other work, simply leave the silver in the borax water, and just before setting the table for dinner, pour away the soapy mixture and rinse the silver with clean cold water, after which wipe carefully with soft canton flannel.

This is a labor-saving plan, and silver washed in this way once a week keeps fresh and bright.

FOR earache, take a bit of cotton, spread it flatly, sprinkle with black pepper, do it up in a wad, dip it in sweet oil and insert in the ear. This is an almost instantaneous relief. The same remedy applied to the cavity of an aching tooth gives immediate relief.

MOTHS can be entirely removed from carpets by wringing a coarse towel out of clear water, spreading it on the carpet, then ironing it dry with a good hot iron. Do this once or twice where the moths are supposed to be. There is no need of pressing hard so that the ply or color of the carpet will be injured. The moths are destroyed by the heat and steam.

WHEN your canary droops and seems ill, particularly if he shows signs of asthma or a cold by a wheezing sound, feed him for a week on boiled bread and milk. Mix bird seed and flax seed and give it ; also strew red pepper plentifully on a piece of salt pork and tie it up in the cage within reach. Give it also a little saffron in its water, now and then.

## Figures and Tables

**Figure f1:**
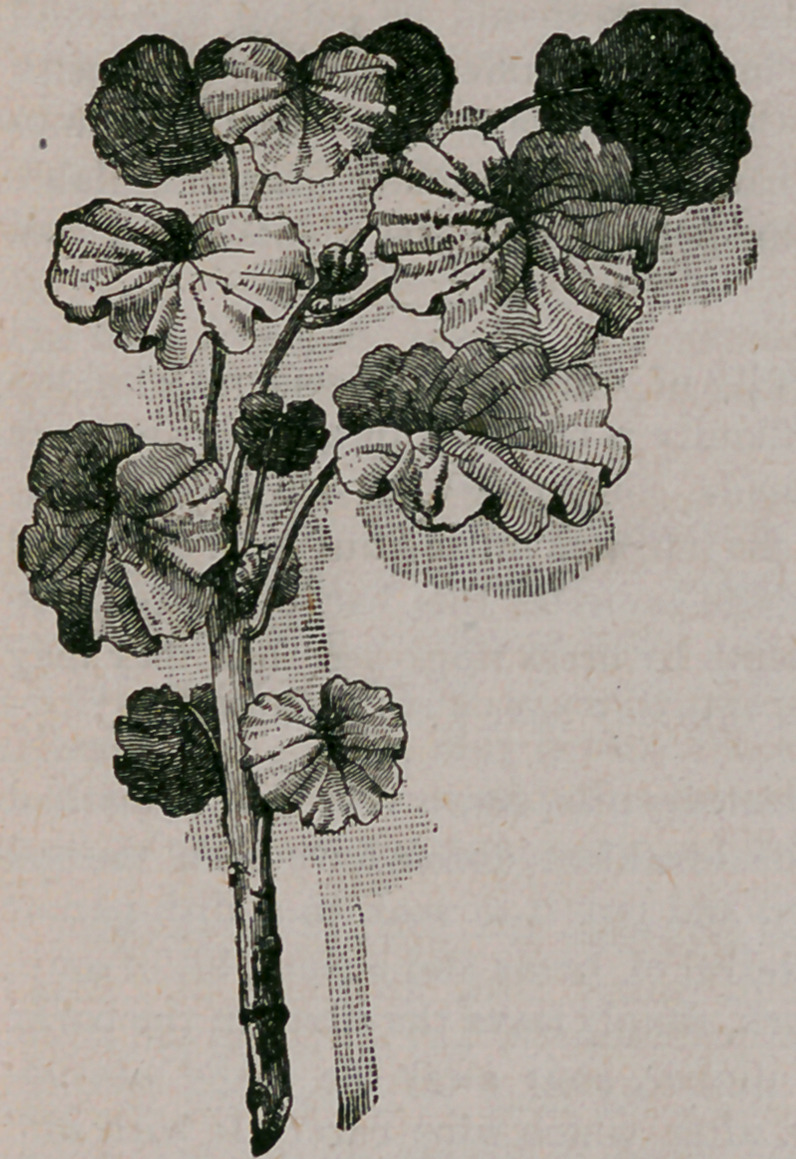


**Figure f2:**